# Significance and mechanisms of perineural invasion in malignant tumors

**DOI:** 10.3389/fonc.2025.1572396

**Published:** 2025-05-12

**Authors:** Mengyao Wang, Niu Pu, Xitong Bo, Fuxiang Chen, Yilong Zhou, Qiong Cheng

**Affiliations:** ^1^ Key Laboratory of Neuroregeneration of Jiangsu and Ministry of Education, Co-Innovation Center of Neuroregeneration, NMPA Key Laboratory for Research and Evaluation of Tissue Engineering Technology Products, Nantong University, Nantong, China; ^2^ Department of Surgery, Nantong Tumor Hospital, Affiliated Tumor Hospital of Nantong University, Medical School of Nantong University, Nantong, China

**Keywords:** malignant tumors, perineural invasion, matastasis, molecular mechanisms, tumor microenvironment, neuro-tumor crosstalk

## Abstract

Cancer remains the second leading cause of death worldwide. Tumor invasion and metastasis pose significant challenges for clinical management. In addition to the traditional pathways of metastasis such as hematologic or lymphatic transmission, perineural invasion (PNI) has become a unique mechanism of metastasis, which is closely associated with neuropathic pain, motor deficits, and poor prognosis. PNI is often observed in malignant tumors of the pancreas, head and neck, gastrointestinal tract, and lungs, and it reflects a unique neurotropic transfer behavior utilizing neural networks. Despite its clinical significance, targeted therapies for PNI are still lacking. This review synthesizes current evidences regarding PNI, elucidates the clinical significance of PNI in tumor metastasis, prognosis, and neurological dysfunction. By integrating the latest advances in multi-omics, we analyzed the potential key molecular pathways and tumor microenvironment drivers of PNI, and proposed future research directions for developing PNI-specific therapies to improve patient outcomes.

## 1 Introduction

**Figure 1:**
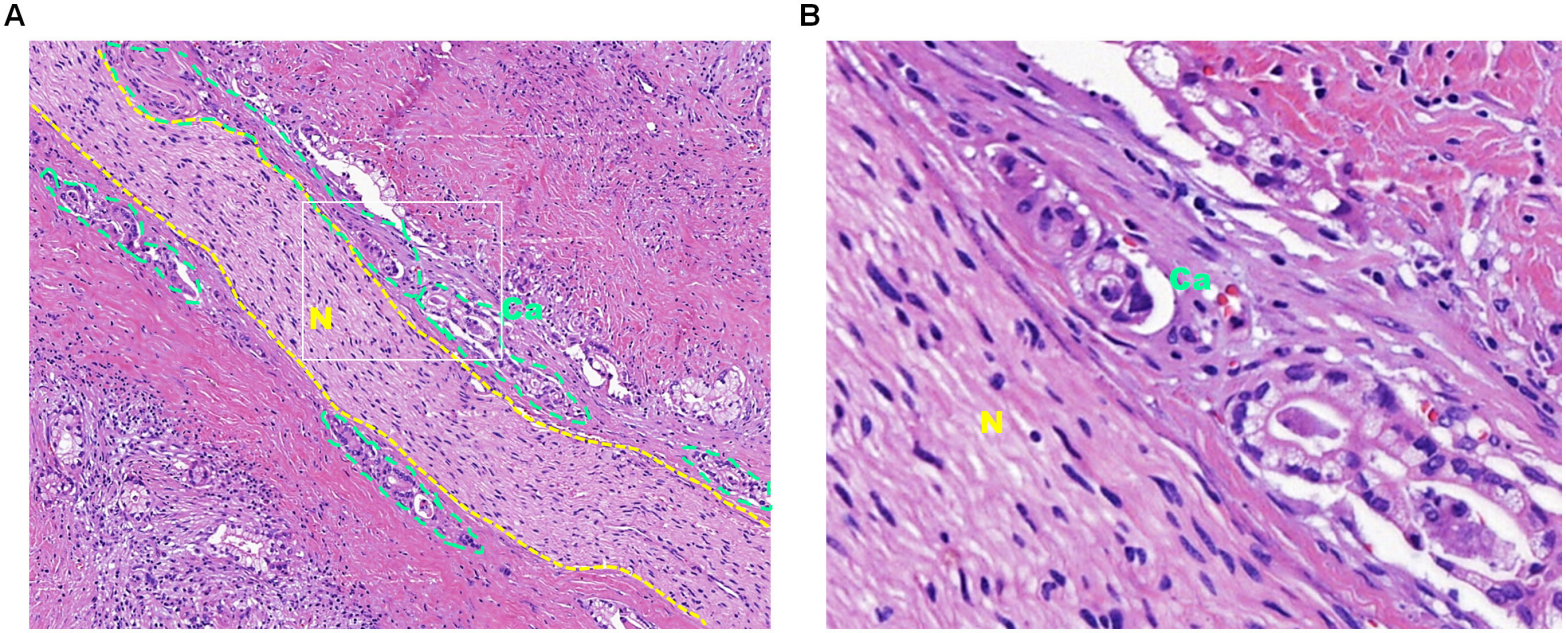
Hematoxylin and eosin staining of pancreatic cancer tissue subjected to perineural invasion. **(A)** Hematoxylin and eosin staining of pancreatic cancer tissue at 10× magnification. The dotted yellow lines outline the nerve tissue, and the dotted green lines outline the cancer cells that invade the nerve. **(B)** Enlarged view of the white box in panel A at 40× magnification, with “Ca” indicating cancer tissue and “N” indicating nerve tissue.

According to data from the World Health Organization, cancer is now the second leading cause of death worldwide (1). The uncontrolled growth of tumors, invasion into surrounding tissues, and their ability to metastasize to distant organs are not only key indicators of malignancy but also pose significant challenges in cancer prevention and treatment (2). In addition to common metastatic routes, such as direct extension, hematogenous spread, lymphatic dissemination, and implantation metastasis (3), perineural invasion (PNI) has emerged in recent years as a distinct and noteworthy mode of metastasis, also known as neurotropic or perineural cancer dissemination. PNI is defined as the involvement of at least 33% of the nerve circumference or the presence of cancer cells in any of the three layers of the nerve sheath (4), and this invasion can cause symptoms such as pain, numbness, muscle weakness, and other neurological dysfunction, all of which can negatively impact treatment outcomes and the patients’ quality of life. PNI is commonly observed in tumors located in areas with rich nerve networks, such as the head and neck, lungs, pancreas, stomach, colon, and rectum (5), and has been recognized as a significant pathological feature of various malignant tumors (6). For example, postoperative pathological examination of pancreatic cancer often shows significant PNI (**Figure 1**).

Despite increasing awareness of PNI, targeted therapies addressing this pathological phenomenon are unavailable. This review aims to clarify the significance of PNI in malignant tumors, including the relationship between PNI and tumor metastasis, prognosis, and neurological dysfunction, summarize the key signaling pathways involved in PNI and the influence of tumor microenvironment on PNI, and propose future research directions.

## 2 Significance of PNI

### 2.1 Impact on tumor metastasis

#### 2.1.1 Differences between PNI and lymphatic, hematogenous metastasis

Initially, PNI was thought to be an extension of lymphatic or hematogenous metastasis. However, the occurrence of PNI in the absence of lymphatic or vascular infiltration indicates that PNI is a distinct pathological entity (7). The differences between PNI and lymphatic, hematogenous metastasis were described in detail in **Table 1**. They differ mechanically, anatomically, and clinically (8–13). PNI places more emphasis on local neural aggression, while lymphatic and hematogenous routes represent regional and systemic dissemination, respectively. Understanding these distinctions can help develop personalized treatment strategies and improving patient outcomes.

**Table 1:** Key differences between PNI and lymphatic, hematogenous metastasis.

Feature	PNI	Lymphatic metastasis	Hematogenous metastasis
Primary Routes	Spreads along nerve trunks or perineurium.	Primary tumor → regional lymph nodes → distant lymph nodes (e.g., breast cancer → axillary nodes; gastric cancer → Virchow’s node).	Disseminates via venous systems to organs such as the lungs, liver, brain, and bones (e.g., colorectal cancer → liver metastases; lung cancer → brain metastases).
Mechanisms	Relies on anatomical proximity; tumor cells interact with the neural microenvironment via neurotrophic factors (e.g., NGF). May involve indirect spread through perineural lymphatic or vascular channels but primarily extends locally along nerve pathways.	Tumor cells are chemotactically recruited to lymphatic vessels by factors like CCL21, entering the lymphatic circulation. Lymph nodes act as “filters” to trap circulating tumor cells, forming metastatic foci.	Tumor cells acquire invasiveness via EMT, penetrate vascular walls, and enter the bloodstream. After lodging in distant organs, metastatic colonies form via mesenchymal-epithelial transition.
Common Cancers	Pancreatic, head/neck, prostate	Breast cancer, melanoma, head and neck cancer, gastric cancer.	Lung cancer, hepatocellular carcinoma, renal cell carcinoma, thyroid cancer.
Symptoms	Neuropathic pain, sensory deficits, motor weakness [e.g., pancreatic cancer-associated back pain (8)].	Painless lymphadenopathy (e.g., neck mass), localized edema [e.g., arm swelling from axillary metastasis in breast cancer (9)].	Organ-specific dysfunction (e.g., jaundice in liver metastasis; pathological fractures in bone metastasis).
Diagnosis	MRI or histopathological examination (microscopic evidence of tumor encircling nerves).	Palpation, ultrasound-guided lymph node biopsy, sentinel lymph node mapping	Imaging (CT, PET-CT), circulating tumor cell detection.
Therapeutic Focus	Wide surgical resection (may sacrifice nerve function), radiotherapy (targeting neural pathways), drugs targeting neurotrophic signaling.	Lymphadenectomy, radiotherapy, chemotherapy (e.g., AC regimen for breast cancer).	Systemic chemotherapy, targeted therapy [e.g. EGFR inhibitors (10)], immunotherapy [e.g. PD-1 inhibitors (11)]
Prognostic Impact	Indicates aggressive behavior with high local recurrence rates [e.g., pancreatic cancer with PNI: 5-year survival <10% (12)].	Lymph node involvement determines staging but may respond to systemic therapy (13).	Advanced disease marker with poor outcomes

PNI, perineural invasion; EMT, epithelial–mesenchymal transition; CCL21, C-C motif chemokine ligand 21; NGF, nerve growth factor; CT, computed tomography; PET/CT, positron emission tomography/computed tomography; EGFR, epidermal growth factor receptor

#### 2.1.2 Main molecular mechanisms of PNI promoting metastasis

PNI is not limited to local invasion; it can also act as a source for distant metastasis. In certain tumor types, PNI may be the sole mechanism of metastatic spread (7). PNI promotes the metastasis of malignant tumors through multidimensional molecular interactions.

Tumor cells that invade nerves tend to have enhanced interactions with the perineuronal blood vessels and lymphatic networks. For example, PNI-positive prostate cancer cells overexpress vascular endothelial growth factor C (VEGFC), promoting lymphatic metastasis of prostate cancer (14). During the PNI process of prostate cancer, cancer cells can induce the formation of abnormal neovascularization networks within the nerve and simultaneously promote vascular invasion (15).

PNI establishes a specialized neuro-tumor interactive microenvironment (16). Within this milieu, neurons and Schwann cells (SCs) release a spectrum of regulatory mediators such as neurotrophic factors (e.g. nerve growth factor (NGF), brain derived neurotrophic factor), chemokines (e.g. C-X3-C motif chemokine ligand 1, ephrin type-A receptor 2, C-C motif chemokine ligand (CCL) 2), axon guidance molecules (e.g. Semaphorin 3D) and neurotransmitters (e.g. glutamate, acetylcholine, dopamine) (17–19). These neural-derived signaling molecules activate corresponding receptors on cancer cells through paracrine signaling, triggering downstream cascades that drive the activation of metastasis-associated signaling pathways (20). **Table 2** summarizes the main molecular mechanisms of PNI promoting tumor metastasis (21–24).

**Table 2:** Main molecular mechanisms of PNI promoting tumor metastasis.

Mechanisms	Involved molecules/pathways
Schwann cell-guided migration along nerve bundles to distant anatomical sites	Laminin, MMP-2/MMP-9 (15, 21)
Enhanced interaction with perineuronal lymphatic/blood networks	VEGF-C, cAMP-PKA (22)
Establishment of neuro-tumor interactive microenvironment	NGF, BDNF, CX3CL1, EPHA2, CCL2, SEMA3D, TrkA, TrkB, RAS-MAPK, PI3K/AKT, JAK2-STAT3, Hippo-YAP/TAZ, Wnt/β-catenin
Neuro-immune crosstalk creates a chronic inflammatory environment that promotes tumor metastasis	Substance P, NK1R, NF-κB, IL-6, TNF-α (23, 24)
Positive correlation between PNI extent and regional metastasis	/

MMP, matrix metalloproteinase; VEGF-C, vascular endothelial growth factor C; cAMP/PKA, cyclic adenosine monophosphate/protein kinase A; CCL21, C-C motif chemokine ligand 21; NGF, nerve growth factor; BDNF, brain derived neurotrophic factor; CX3CL1, C-X3-C motif chemokine ligand 1; EPHA2, ephrin type-A receptor 2; SEMA3D, semaphorin 3D; TrkA, tyrosine receptor kinase A; TrkB, tyrosine kinase receptor B; RAS/MAPK, RAS-mitogen-activated protein kinase; PI3K/AKT, phosphatidylinositol 3-kinase/protein kinase B; JAK2/STAT3, Janus kinase 2/signal transducers and activators of transcription 3; NK1R, neurokinin-1 receptor; NF-κB, nuclear factor-kappaB; TNF-α, tumor necrosis factor alpha; IL-6, interleukin 6

### 2.2 Prognostic implications

PNI is closely associated with the prognosis of patients with malignant tumors (25, 26). In periocular skin squamous cell carcinoma, the higher the degree of PNI, the greater the risk of tumor recurrence, regional metastasis, and high mortality (27). In oral squamous cell carcinoma, PNI predicts the risk of lymph nodes occult metastasis (28). In pancreatic carcinoma, PNI-positive patients had an increased risk of liver metastasis (29). In patients with resectable pancreatic cancer undergoing preoperative chemotherapy with gemcitabine, PNI can serve as an important indicator for evaluating surgical outcomes and recurrence, and it is a significant independent factor related to postoperative disease-free survival (30). The incidence of PNI is high among patients with head and neck cancer, particularly those receiving radiotherapy, where an elevated pretreatment PNI is significantly correlated with poorer overall survival, distant metastasis-free survival, and progression-free survival (31). Approximately 20%-31% of patients with intrahepatic cholangiocarcinoma (ICC) exhibit PNI, which is associated with reduced overall and recurrence-free survival, as well as an increased tendency for immunosuppressive metastasis. After receiving adjuvant therapy with TEGIO, GEMOX, or capecitabine, patients with ICC complicated by PNI have improved survival, indicating a favorable response to adjuvant chemotherapy (32). In patients who have undergone potentially curative surgery for ICC, PNI is a strong independent predictor of tumor recurrence and long-term survival; therefore, it should be routinely assessed in clinical practice (33).

### 2.3 Neurological complications

#### 2.3.1 Cancer-related neuropathic pain mechanism

PNI is a key factor in the development of cancer pain, and pain itself can also serve as a predictor of PNI (34, 35). The mechanisms by which PNI contributes to cancer pain are complex and involve various factors, such as nerve compression, neuroinflammation, and neuronal damage (36). Neuropathies resulting from PNI, along with the upregulation of cancer-associated fibroblasts (CAFs), tumor-associated macrophages (TAMs), neurotrophic factors, and substance in the tumor microenvironment, can exacerbate neuropathic pain (34, 37). Additionally, PNI can lead to abnormalities in nerve conduction, resulting in the amplification or abnormal transmission of pain signals (38). Targeting signaling pathways associated with PNI, such as NGF/tyrosine receptor kinase A (TrkA), may effectively alleviate cancer pain and improve treatment outcomes for tumors (39).

#### 2.3.2 Neurological dysfunction syndromes in PNI-associated cancers

PNI disrupts nerve structure and impairs nerve conductive function, leading to muscle weakness or paralysis in the areas innervated by the affected nerves. In patients with advanced prostate cancer, tumors may directly invade the lumbosacral plexus, resulting in radiculopathy of the lower limbs, characterized by weakness, numbness, and loss of control over bowel or bladder function (40, 41). Head and neck cancers, particularly squamous cell carcinoma and adenoid cystic carcinoma, can directly invade the facial nerve, resulting in slowly progressive facial paralysis (42, 43). PNI may also lead to autonomic nerve dysfunction (44). PNI of ICC primarily affects the sympathetic nerve (32), and inhibiting sympathetic nerve activity can improve prognosis and prolong survival in these patients (45). Additionally, suppressing sympathetic signaling in patients with pancreatic ductal adenocarcinoma (PDAC) may increase the efficacy of anticancer drugs, reduce the tumor burden, and prolong survival (46).

## 3 Molecular pathways involved in PNI

PNI is a bidirectional process that involves interactions between cancer cells and nerves rather than being triggered solely by the unidirectional invasion of nerves by cancer cells (47). This bidirectional cellular interplay is governed by evolutionarily conserved molecular pathways that orchestrate both neurotropic attraction and tumor advancement. Systematic investigation of these signaling cascades serves dual purposes: elucidating the mechanistic basis of PNI while simultaneously identifying therapeutically actionable targets for clinical intervention.

### 3.1 NGF/TrKA signaling pathway

NGF binds to the high-affinity receptor TrkA and the low-affinity receptor p75^NTR^, playing crucial roles not only in neurodevelopment but also in pain perception, immune regulation, and the occurrence and progression of tumors in adults (48–50). Increased expression of NGF and its receptors in tumor cells is associated with a greater frequency and severity of PNI (51, 52). For example, in cholangiocarcinoma, activation of the NGF/TrkA pathway promotes the proliferation and invasion of cancer cells. Elevated levels of NGF bind to TrkA in nerve sheaths, facilitating the development of PNI (50, 53). In the extracellular matrix (ECM) of ICCs, NGF expression is upregulated, which activates TrkA receptors on tumor cells through a paracrine mechanism, thereby promoting PNI (54). In pancreatic cancer, the NGF/TrkA pathway enhances PNI by promoting the Warburg effect and upregulating the expression of miR-21-5p (52). Pancreatic cancer cells typically exhibit high levels of NGF, and knocking down TrkA can inhibit PNI in these cells (55). Additionally, NGF acts as a key driver of neuroinflammation in the tumor microenvironment of pancreatic cancer, and its inhibition can significantly reduce tumor-associated PNI and alleviate cancer-related pain (56, 57). Furthermore, the low-affinity NGF receptor p75^NTR^ can act as a chemotactic agent, attracting cancer cells toward nerve tissues and thereby promoting PNI (51). Additionally, NGF released by SCs may facilitate the migration of glial cells during the process of PNI (58, 59).

### 3.2 Notch signaling pathway

The Notch signaling pathway is a highly conserved mechanism that plays a critical role in various physiological processes (60). Aberrant activation of this pathway is closely associated with PNI. In oral squamous cell carcinoma, strong expression of Notch1 and Notch4 is linked to PNI (61, 62). Similarly, elevated expression of Notch4 is closely related to PNI in salivary gland adenoid cystic carcinoma (63, 64). Additionally, the expression of the Notch ligand DLL4 and the γ-secretase complex component nicastrin is also associated with PNI (65, 66). Moreover, the Notch signaling pathway is a key driver of epithelial–mesenchymal transition (EMT) induced by transforming growth factor-beta, which promotes tumor invasion and metastasis by regulating molecules such as Twist, Snail, and E-cadherin (67–69). Components of the Notch pathway, particularly Notch4, are highly expressed in various tumors complicated by PNI, making them potential biomarkers for predicting PNI and poor prognosis. Targeting the Notch pathway, such as by silencing Notch4 with siRNA, can effectively inhibit the PNI capabilities of tumor cells, indicating its potential as a therapeutic target.

### 3.3 c-Jun N-terminal kinase signaling pathway

JNK, a member of the mitogen-activated protein kinase (MAPK) family, can be activated by various extracellular signals, such as growth factors and inflammatory mediators (70, 71). The JNK signaling pathway plays a crucial role in tumor-associated PNI. In PDAC, glial cell line-derived neurotrophic factor (GDNF) secreted by SCs can promote PNI and metastasis by activating the JNK/ZEB1/EMT axis (17). GDNF expression is significantly elevated in oral cancer tissues, and GDNF treatment enhances the phosphorylation of extracellular signal-regulated kinases (ERK), p38, and JNK, which increases the DNA-binding activity of AP-1 and increases the expression of matrix metalloproteinases (MMPs). This, in turn, increases the migratory capacity of tumor cells. Notably, the inhibition of MAPK or AP-1 effectively reduces GDNF-induced PNI (72). In salivary gland adenoid cystic carcinoma, the phosphorylation levels of JNK and p38 are closely associated with PNI. Elevated levels of phosphorylated p38 are considered an independent prognostic factor for poor prognosis, whereas JNK activation is correlated with tumor invasiveness (73). In rectal cancer, serine protease inhibitor Kazal type 1 (SPINK1) enhances the proliferation and migration of cancer cells by activating epidermal growth factor receptor-downstream ERK, p38, and JNK signaling pathways. Elevated SPINK1 expression is obviously associated with the PNI and is a crucial prognostic indicator for overall survival and disease-free survival (74). BAP1 is a tumor suppressor, and its downregulation is linked to the invasiveness of ICC. BAP1 exerts its tumor-suppressive effects by inhibiting the JNK/c-Jun pathway, suggesting that the JNK signaling pathway could serve as a potential therapeutic target in ICC (75, 76).

Notably, JNK1 and JNK2 play distinct functional roles in various cancers. Specific inhibition of JNK2 increases the oncogenic potential of pancreatic cancer cells, whereas inhibition of JNK1 has the opposite effect (77). These findings suggest that targeted regulation of JNK isoforms may provide new strategies for cancer therapy.

### 3.4 Wnt signaling pathway

The Wnt signaling pathway is a crucial regulator of tissue homeostasis and is closely associated with cancer, playing a significant role in PNI. This pathway can be categorized into two types: β-catenin-dependent (canonical) signaling and β-catenin-independent (noncanonical) signaling. The canonical Wnt signaling pathway stabilizes and promotes the accumulation of β-catenin when Wnt ligands bind to Frizzled and low-density lipoprotein receptor-related protein, allowing β-catenin to enter the nucleus and initiate the transcription of target genes. In contrast, the noncanonical Wnt signaling pathway regulates cell polarity and movement through different mechanisms (78).

Both the canonical and noncanonical Wnt signaling pathways promote tumor PNI in a highly tissue-specific manner. In head and neck squamous cell carcinoma, a secreted inhibitor of the Wnt signaling pathway DKK1 is upregulated, which can promote PNI by activating the phosphatidylinositol 3-kinase/protein kinase B (PI3K/AKT) signaling pathway (79). In gastric cancer, increased expression of USP15 triggers the nuclear translocation of β-catenin, increasing cell proliferation and promoting PNI (80). In pancreatic cancer, the miRNA tRF-19-PNR8YPJZ activates the Wnt pathway, increasing cell proliferation and metastasis and promoting PNI (81). In advanced metastatic bladder cancer, the upregulation of SFRP2 promotes EMT and promote PNI (81). In prostate cancer, dysfunction of primary cilia leads to enhanced Wnt signaling, promoting the occurrence of PNI (82). Furthermore, both canonical and noncanonical Wnt signaling can increase tumor invasiveness and promote PNI by regulating EMT-related transcription factors such as Snail2 and ASPP2 (83, 84).

### 3.5 Emerging mechanisms unveiled by multi-omics profiling

Recent advances in single-cell RNA sequencing (scRNA-seq), spatial transcriptomics, and proteomics have significantly advanced our understanding of PNI. These technologies collectively unravel the molecular complexity of PNI by dissecting cellular heterogeneity, spatial dynamics, and functional interactions.

scRNA-seq has identified tumor subpopulations with distinct neurotropic properties. In prostate cancer, basal/intermediate epithelial cells expressing polymeric immunoglobulin receptor, MMP-7, and anterior gradient 2 were associated with PNI progression, while trajectory analysis suggested luminal cells as potential precursors (85). Similarly, in distal cholangiocarcinoma, hypoxia-prone NEAT1^+^ tumor cells and hypermetabolic GFAP^+^ dedifferentiated SCs formed a lactate-high mobility group box-1 axis driving invasion (86). SC plasticity, characterized by upregulated secretory proteins (SCG2, VGF), was also observed in pancreatic cancer, mirroring nerve injury responses (87). scRNA-seq further revealed immune microenvironment shifts, such as CD161^+^CD8^+^ T cells in PDAC, which exhibited dual cytotoxic and immunosuppressive roles correlated with survival (88).

Spatial transcriptomics has mapped molecular gradients and immune landscapes at nerve-tumor interfaces. In oral squamous cell carcinoma, nerves near tumors showed enriched stress response (HSPA1A) and growth signaling (EGFR), fading with increasing distance (89). PDAC studies highlighted axon guidance pathways and endocannabinoid metabolism in PNI, alongside immune “cold” niches marked by PD-L1 upregulation (90). Spatial profiling also enabled practical innovations, such as MHC-II-targeted peptide probes in adenoid cystic carcinoma (91), which dynamically labeled SCs in PNI regions for intraoperative guidance.

Proteomics validated pathway activities and post-translational modifications underlying PNI. In PDAC, VGF-mediated neurite extension was confirmed *in vitro* (87), while cholinergic signaling in PNI suppressed CD8^+^ T-cell recruitment via CCL5 downregulation, promoting Th2 polarization (92). Metabolic reprogramming, such as lactate-induced SC dedifferentiation in distal cholangiocarcinoma (86), further underscored microenvironmental crosstalk. Epigenetic proteomics in head and neck cancer linked DNA hypermethylation (CDH1, TIMP3) and histone modifications (H3K27me3) to EMT and PNI progression (93).

PNI process is regulated by chemokines, growth factors, cell adhesion and epigenetics (6, 18, 94–116). A thorough understanding of the molecular mechanisms involved is essential for developing targeted interventions against PNI (**Table 3**).

**Table 3:** Molecular mechanisms involved in PNI.

Mechanisms	Key molecules/pathways	Functional description	Associated cancers	Potential therapeutic targets
Cell-Cell Interactions	NCAM	Mediates tumor-neural cell adhesion to promote invasive migration	Pancreatic, Head and Neck, Prostate cancers	NCAM inhibitors (94)
	L1CAM	Facilitates tumor-Schwann cell interaction to form a pro-invasive microenvironment	Pancreatic cancer, Glioma	L1CAM blockers (e.g., cetuximab analogs) (95)
	Integrins	Binds perineural matrix to enhance migratory capacity	Various solid tumors	Integrin antagonists (e.g., Cilengitide) (96)
Chemokines &Axon Guidance	GDNF/RET Pathway	GDNF activates RET receptor to drive neural tracking, activating PI3K/AKT and MAPK pathways	Pancreatic, Prostate cancers	RET inhibitors (e.g., Selpercatinib) (6)
	NGF/TrkA Pathway	NGF via TrkA activates Ras-MAPK and PI3K pathways, promoting proliferation and invasion	Head and neck, Prostate, Breast cancers	TrkA inhibitors (e.g., Larotrectinib) (97)
	CX3CL1-CX3CR1 Axis	CX3CL1 (Fractalkine) recruits CX3CR1^+^ tumor cells to nerves, suppressing apoptosis	Gastric, Pancreatic cancers	CX3CR1 antagonists (e.g., AZD8797) (98)
	Semaphorins (SEMA3D/3C/4D/3A)	SEMA3D/3C activates PlexinD1/A2 to promote invasion; SEMA4D activates RhoA/ROK for cytoskeletal remodeling	Pancreatic cancer	SEMA3D knockdown (18, 99, 100)
Growth Factors &Matrix Remodeling	TGF-β/SMAD Pathway	Induces EMT, activates Schwann cells, and promotes MMP-mediated matrix degradation	Pancreatic, Cholangiocarcinoma	TGF-β inhibitors (e.g., Galunisertib) (101, 102)
	VEGF/Neuropilin Axis	Promotes perineural angiogenesis and directly stimulates tumor invasion	Breast, Pancreatic, Head and Neck cancers	VEGF inhibitors (e.g., Bevacizumab) (103)
	MMPs	Degrades perineural matrix (collagen, laminin) to enable invasion	Various solid tumors	MMP inhibitors (e.g., Marimastat) (104)
Neuro-Tumor Crosstalk	Acetylcholine/M3 Receptor	Activates AKT signaling pathway to drive tumor cell invasion	Cholangiocarcinoma	Cholinergic antagonists (e.g., Atropine) (105)
	Adrenaline/β-Adrenergic Receptor	Activates STAT3 pathway to promote invasion; Activates cAMP/PKA pathway to enhance invasiveness	Pancreatic, Breast cancers	β-blockers (e.g., Propranolol) (106, 107)
	Schwann Cell-Secreted IL-6	Promotes tumor survival and inflammation via IL-6/STAT3 signaling	Pancreatic cancer	IL-6R antibodies (e.g., Tocilizumab) (108)
Epigenetic Regulation	DNA Methylation (e.g., FAM134B, ZNF677, ABAF-1)	Hypermethylation of tumor suppressors promotes invasive phenotypes	Colorectal, Gastric, Pancreatic cancers	Demethylating agents (e.g., 5-Azacytidine) (109–111)
	miRNAs (e.g., miR-21, miR-211-5p)	Generates RNA-induced silencing complex to orchestrate oncogene and anti-oncogene	Various malignant cancers	miRNA mimics/antagonists (112–114)
Immune Microenvironment	CCL2-CCR2 Axis	CCL2 released from Schwann cells recruits TAMs to promote perineural inflammation and tumor invasion	Pancreatic, Breast cancers	CCR2 inhibitors (115)
	PD-L1	Induces immune evasion in perineural regions	Gastric cancer	PD-L1 antibodies (e.g., Atezolizumab) (116)

L1CAM, L1 cell adhesion molecule; NCAM, neural cell adhesion molecule; GDNF, glial cell line-derived neurotrophic factor; RET, rearranged during transfection; TrkA, tyrosine receptor kinase A; CX3CL1, C-X3-C motif chemokine ligand 1; SEMAs, semaphorins; TGF-β, Transforming growth factor beta; SMAD, Suppressor of mother against decapentaplegic; VEGF, vascular endothelial growth factor; MMPs, Matrix metalloproteinases; AKT, protein kinase B; STAT3, signal transducers and activators of transcription 3; IL-6, interleukin 6; miRNAs, MicroRNAs; CCL2, C-C motif chemokine ligand 2; CCR2, C-C chemokine receptor type 2; PD-L1, Programmed death-ligand 1.

## 4 Tumor microenvironment constituents orchestrating PNI

The tumor microenvironment (TME) comprises heterogeneous components, including vascular/lymphatic networks, ECM, stromal/immune cells, secretory factors, noncoding RNAs, and extracellular vesicles, that collectively establish a pro-metastatic niche (117). Within this ecosystem, SCs, the principal myelinating glia of peripheral nerves, emerges as pivotal orchestrators of PNI. Beyond SCs, CAFs, TAMs, and infiltrating T cells further potentiate PNI via autocrine-paracrine signaling axes, highlighting the multicellular cooperativity with TME (118). The following sections describe how they drive PNI from the perspective of non-tumor cell components of TME.

### 4.1 SCs

**Figure 2:**
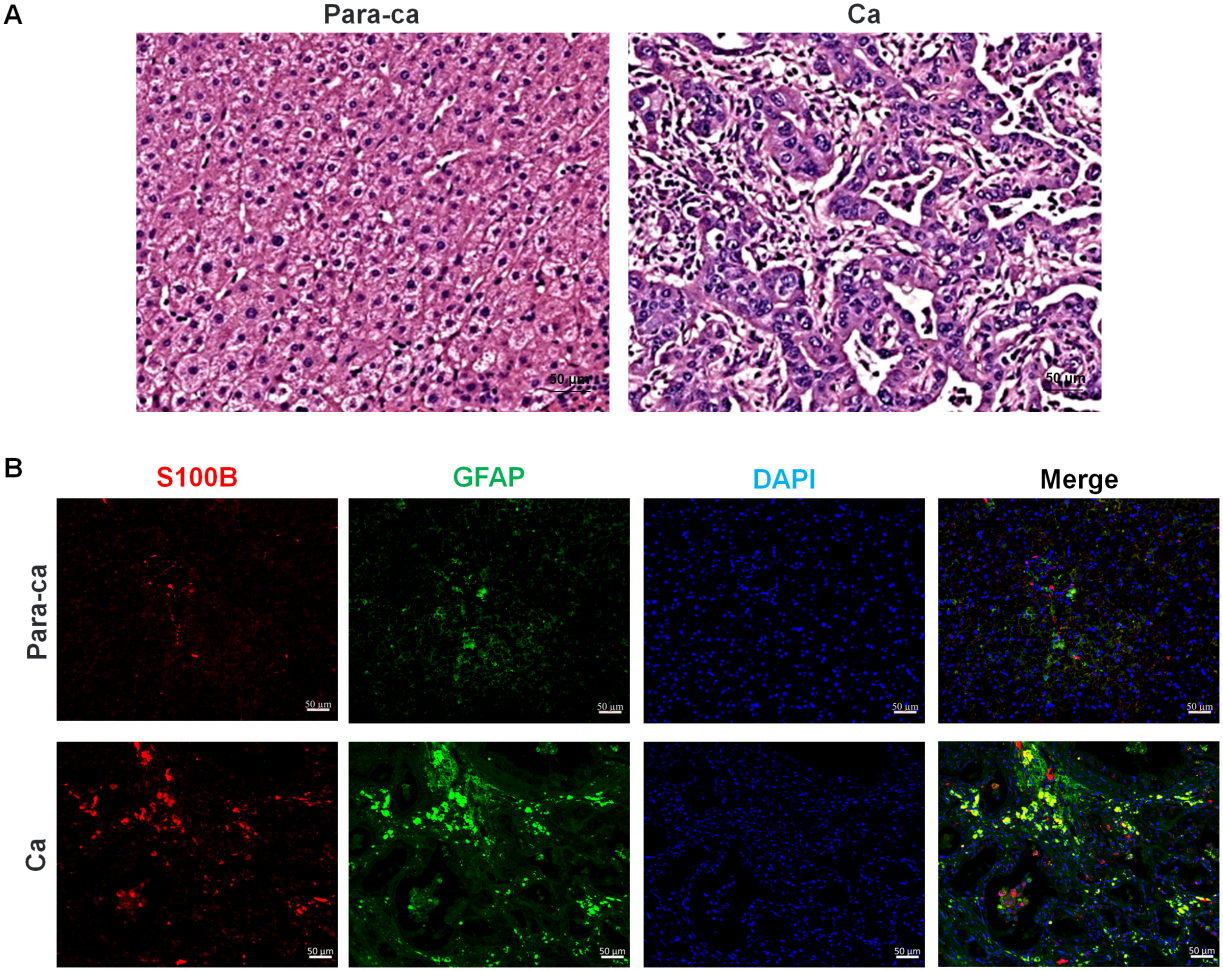
Pathological section staining of intrahepatic cholangiocarcinoma (ICC) with para-carcinoma and carcinoma tissues. **(A)** H&E staining of para-carcinoma and carcinoma tissues. **(B)** S100B and GFAP immunofluorescence staining of para-carcinoma and carcinoma tissues. Red, S100B; Green, GFAP; Blue, DAPI, and Merge is their overlap. Bar=50 μm.

Within the tumor microenvironment, SCs act as bridges at the tumor–nerve interface, playing a role in both the nerve supply to tumors and the invasion of tumors into adjacent nerves (119, 120). In tumors with high rates of PNI, such as ICC, the expression of SC markers such as S100B and glial fibrillary acidic protein (GFAP) is obviously elevated (**Figure 2**).

SCs derived from neural crest precursors differentiate postnatally into two functionally distinct subtypes: myelinating SCs, which insulate large axons via myelin proteins (MBP, MPZ), and non-myelinating SCs (including Remak SCs) that ensheath small-caliber axons while expressing markers such as p75NTR and GFAP (121, 122). This developmental plasticity is recapitulated during nerve injury, where mature SCs dedifferentiate into a repair phenotype characterized by c-Jun/Sox2 upregulation and myelin gene suppression, promoting nerve regeneration (123). However, this process is hijacked by cancers to facilitate PNI (124). SCs guide cancer migration via neural cell adhesion molecule 1-dependent protrusion formation, enabling cancer dispersion along nerves. This mechanism is similar to the repair phenotype of SCs after nerve injury, while in cancer, it directly leads to the invasion of nerve tissue by cancer cells (125). In PDAC, c-Jun-activated SCs form tumor-activated Schwann cell tracks that enhance cancer cell motility and promote PNI. However, the use of a c-Jun N-terminal kinase inhibitor (e.g., SP600125) for SCs can effectively reduce the activation of pro-invasive SC (126). PDAC-derived extracellular vesicles upregulate p75NTR in SCs, which is a biomarker closely related to PNI (127). Similarly, colon cancer-derived exosomal miR-21-5p suppresses Von Hippel-Lindau expression in SCs, stabilizing hypoxia-inducible factor-1α to elevate NGF secretion, which reciprocally activates ERK signaling and promotes PNI in cancer cells (128).

However, not all reprogrammed SCs exhibit tumor-promoting functions; reprogrammed SCs may also exert tumor-suppressive effects. Emerging evidence indicates that pancreatic SCs undergo adaptive reprogramming during early carcinogenesis to support protective anti-tumor neuronal responses. Using sparse genetic labeling, the study demonstrated that SCs in early PDAC overexpress neurotrophic factors, particularly GDNF. This altered SCs phenotype drives enhanced sympathetic neuronal projections, thereby establishing an inhibitory microenvironment that suppresses cancer progression (129).

ScRNA-seq has unveiled the dynamic heterogeneity of cellular subpopulations within the TME. SCs exhibit functional heterogeneity in the TME and regulate tumor progression through interactions with immune and stromal cells. In PNI-positive distal cholangiocarcinoma, five SCs subpopulations have been identified, with GFAP^+^ dedifferentiated SCs displaying hypermetabolic characteristics. Lactate in hypoxic TME induces GFAP-dedifferentiation in SCs, which promotes cancer cell invasion and progression via upregulation of HMGB1 (86). In vestibular schwannomas, tumor-associated SCs comprise “repair-like” and MHC-II antigen-presenting subpopulations that actively recruit macrophages through CSF1 signaling, mimicking nerve injury repair processes to drive tumor growth. Tumors exhibiting this “injury-like” phenotype demonstrate larger volumes and activation of nerve repair-associated transcription factors (130). In plexiform neurofibromas, NF1 deficiency drives SCs differentiation into five subpopulations, including a Schwann cell progenitor-like population characterized by elevated expression of Postn and CD74. These SCs aberrantly interact with immune/stromal cells through PROS1-AXL, FGF-FGFR, and MIF-CD74/NF-κB pathways, establishing a TME dominated by immune and stromal components, with conserved mechanisms across murine and human models (131). Spatial mapping of neuroblastomas reveals that a subset of malignant cells adopts mesenchymal states sharing molecular features with SCs precursors, while spatial compartmentalization segregates malignant cells from immune infiltrates. Epigenetic regulation, particularly DNA methylation patterns, correlates with clinical outcomes (132).

Mechanistically, the functional heterogeneity of SCs within the TME manifests through: i) Differentiation into distinct subpopulations, including *repair-like*, *antigen-presenting*, and *progenitor-like* phenotypes; ii) Recruitment of immune cells or augmentation of sympathetic neuronal activity via secretion of factors such as CSF1, GDNF, and MIF; iii) Formation of a pro-tumorigenic microenvironment through evolutionarily conserved signaling pathways (e.g., CD74-NF-κB) that mediate crosstalk with immune and stromal cells. Therapeutically, potential strategies include targeting SC-driven immune recruitment (e.g., CSF1 blockade), dysregulated signaling pathways (e.g., CD74 and AXL inhibition), or epigenetic modulation. Furthermore, the association between SC precursor-like cells and tumor spatial heterogeneity highlights novel opportunities for modulating the TME.

### 4.2 CAFs

In the tumor microenvironment, CAFs play a central role and exhibit high heterogeneity. They can originate from fibroblasts in adjacent tissues, transdifferentiated cells within the tumor, bone marrow-derived mesenchymal cells, or precursor cells derived from adipocytes, endothelial cells, mesothelial cells, or pericytes (133–135). Single-cell sequencing has categorized CAFs into several subgroups, including myofibroblastic CAFs, inflammatory CAFs, immune-regulatory CAFs, antigen-presenting CAFs, vascular CAFs, and metabolic CAFs. These subgroups are conserved across different organs, cancer subtypes, and species but also present some organ-specific characteristics (136).

The heterogeneity of CAFs leads to diverse functions, including the promotion of cancer cell growth, angiogenesis, ECM remodeling, and the recruitment of suppressive immune cells. In some cases, they may even exert tumor-suppressive effects (137). CAFs can alter the tumor microenvironment through the secretion of growth factors, cytokines, and matrix metalloproteinases, thereby facilitating tumorigenesis and metastasis. A previous study revealed that CAFs are significantly associated with PNI in patients with breast cancer and indicate a poor prognosis (138). Coculturing prostate cancer cells with CAFs enhances the expression of critical component of the Hippo signaling pathway, Yes-associated protein 1, which in turn elevates the secretion of NGF and promote the occurrence of PNI in cancer cells (139). Additionally, CAFs drive PNI in pancreatic cancer cells through the SOX4/MMP11 signaling axis (140). Aberrant activation of SOX4 facilitates the transition of inflammatory CAFs to myofibroblastic CAFs, further increasing the expression of MMP11. Myofibroblastic CAFs also contribute to EMT, thereby enhancing the invasive capacity of pancreatic cancer cells and promoting PNI (140).

### 4.3 TAMs

TAMs are critical components of the tumor microenvironment, which originate from circulating monocytes and resident tissue macrophages. Tumor-derived cytokines actively recruit TAMs to the tumor site. TAMs are important sources of epithelial growth factor within tumor tissue (141) and can also produce a variety of other cytokines, including platelet-derived growth factor, transforming growth factor beta-β, hepatocyte growth factor, and basic fibroblast growth factor (bFGF), thus promoting tumor cell proliferation and survival, acting as “signal transducers” in the interactions between tumors and neurons.

TAMs are closely associated with the PNI of tumors, primarily influencing cancer progression by promoting EMT (142). Prior to PNI, TAMs accumulate near tumor cells and assist in the polarization and migration of these cells through the GDNF/RET pathway. The number of TAMs surrounding the tumor-invading nerves is greater than that surrounding normal nerves (34). In patients with pancreatic cancer accompanied by PNI, the infiltration of TAMs in tumor tissue is significantly greater than that in patients without PNI (143).

TAMs can also collaborate with other cells in the tumor microenvironment to promote PNI. They can stimulate CAFs to produce leukemia inhibitory factor and SLIT2, inducing the plasticity of dorsal root ganglia (DRGs) and facilitating cancer cell PNI through the JAK/STAT3/AKT and N-cadherin/β-catenin pathways (135). Additionally, TAMs can activate pancreatic stellate cells (PSCs) and promote PNI via cholinergic signaling (144). In PDAC, TAMs can activate PSCs through the bFGF/PI3K/Akt/c-myc/GFAP pathway. PSCs can secrete IL-33, which recruits macrophages into the perineural environment, promoting the M2 polarization of macrophages and, consequently, tumor growth. The positive feedback loop between SCs and TAMs involving bFGF and IL-33 is essential in the process of PNI in PDAC (145). Furthermore, CCR2-expressing inflammatory monocytes are preferentially recruited to PNI sites by CCL2 released from SCs, where they differentiate into macrophages that promote PNI through the release of cathepsin B (115).

### 4.4 T cells

T cells, as important immune cells, play crucial roles in the tumor microenvironment, which can not only exert antitumor effects, but also be suppressed by tumor cells and their microenvironment. The main types of T cells include (i) effector T cells (CD8^+^ T cells), which induce apoptosis in tumor cells by recognizing tumor-specific antigens and releasing cytotoxic molecules such as perforin and granzyme; (ii) helper T cells (CD4^+^ T cells), which enhance the immune response by activating B cells, macrophages, and dendritic cells through the secretion of cytokines such as IL-2 and IFN-γ; and (iii) regulatory T cells, which play important roles in maintaining immune tolerance and suppressing excessive immune responses. In the tumor microenvironment, regulatory T cells typically inhibit the activity of CD8^+^ T cells, aiding tumors in evading immune surveillance.

PNI is associated with T-cell infiltration. In patients with serous ovarian cancer, the severity of PNI is negatively correlated with the infiltration of CD4^+^ T cells in tumor tissue (146). Severe PNI can lead to an immunosuppressive microenvironment in PDAC, which is associated with elevated levels of acetylcholine (Ach) during severe PNI. Increased Ach levels inhibit the expression of CCL5 through HDAC1, impairing the ability of pancreatic cancer cells to recruit CD8^+^ T cells. Ach also directly suppresses the production of IFN-γ by CD8^+^ T cells in a dose-dependent manner and promotes the differentiation of Th2 cells (92). In primary cutaneous squamous cell carcinoma with PNI, infiltrating lymphocytes are predominantly CD8^+^ T cells, which exhibit a relatively high frequency of immune checkpoints such as PD-1, CTLA-4, and Tim-3. These immune checkpoints inhibit T-cell activity through various mechanisms, allowing tumor cells to evade the surveillance of the immune system (147). Additionally, in head and neck squamous cell carcinoma with PNI, sensory nerves can directly interact with the adaptive immune system by releasing calcitonin gene-related peptides, which reduces the number of CD4^+^ T cells in the tumor microenvironment while activating CD8^+^ T cells, thereby accelerating tumor growth (148).

## 5 Conclusion and future perspectives

Although significant progress has been made in understanding the role of neuro-tumor crosstalk in tumor progression, many uncertainties remain regarding the specific mechanisms by which cancer regulates nerves. These include neurogenesis and nerve remodeling in the tumor microenvironment, as well as the development of PNI. Future research should focus on the following areas.

### 5.1 Bidirectional mechanism of PNI

PNI is both the process by which tumors invade nerves and the mechanism through which nerves promote tumor cell behavior. This interaction relies on a range of molecular mediators, including neurotrophic factors, chemokines, cell adhesion molecules, axon guidance cues, neurotransmitters, neuropeptides, and matrix metalloproteinases. A more detailed understanding of the specific roles of these mediators in PNI could offer valuable insights for developing targeted therapies. Future research should prioritize multi-omics integration to map spatiotemporal PNI dynamics, leveraging AI for predictive modeling and therapeutic optimization.

### 5.2 Effect of the tumor microenvironment on PNI

The tumor microenvironment plays a crucial role in PNI. The complex interactions among SCs, immune cells, mesenchymal stem cells, and fibroblasts create the necessary conditions for the initiation and progression of PNI. Targeting neurotrophic axes (GDNF/RET, NGF/TrkA) and metabolic pathways (e.g., lactate transport) may disrupt nerve-tumor crosstalk, while reversing cholinergic immunosuppression or enhancing CD161^+^CD8^+^ T-cell cytotoxicity could reshape immune responses. Studying the interactions between different cell types in the tumor microenvironment and exploring approaches to modulate the tumor microenvironment to inhibit PNI could provide new strategies for cancer treatment.

### 5.3 Discovery of new therapeutic targets

In-depth investigations of the molecules and signaling pathways involved in PNI are crucial for identifying new therapeutic targets. The reliance of cancer cells on nerves for their oncogenic phenotype provides a foundation for targeting these mechanisms. Approaches such as surgery or drugs offer significant translational potential in cancer neuroscience. For example, chemical pancreatic denervation in patients with nonresectable pancreatic cancer has demonstrated potential for pain relief and improved survival (120). However, these denervation strategies may be accompanied by severe side effects, including persistent diarrhea and sexual dysfunction. Future research should focus on optimizing efficacy while minimizing these adverse effects. Clinically, spatial omics-based classifiers (e.g., nerve-tumor distance) and 3D models will refine diagnostics and drug testing. These efforts promise to translate mechanistic insights into therapies for PNI-prone malignancies, ultimately improving patient outcomes.

### 5.4 Development of specific *in vivo* and *in vitro* models of PNI

In-depth research on PNI depends on improving existing experimental models and developing specialized models to help researchers investigate the roles of different cell types in PNI, the tumor microenvironment factors that influence PNI, and phenomena such as immune evasion and inflammatory responses in the tumor microenvironment. Particular attention should be given to the pathological changes and molecular mechanisms occurring in the very early stages of PNI, as well as during the phase of harmful hypersensitivity reactions. The development of specialized PNI models can also aid in identifying potential biomarkers and, when combined with cutting-edge biotechnologies (such as multi-omics and super-resolution imaging), can facilitate the development of new diagnostic tools, ultimately, enabling early intervention in PNI and increasing the effectiveness of cancer therapies.

In conclusion, a deeper understanding of the biological characteristics of PNI not only helps reveal the mechanisms of cancer progression but also opens new avenues for developing novel treatment strategies. Ongoing research in this field will provide a crucial foundation for the early diagnosis and treatment of cancer.
